# Artificial Stupidity: Data We Need to Make Machines Our Equals

**DOI:** 10.1016/j.patter.2020.100021

**Published:** 2020-05-08

**Authors:** Michaël Trazzi, Roman V. Yampolskiy

**Affiliations:** 1 42 France, Paris, France; 2 University of Louisville, Computer Science and Engineering, Louisville, KY, USA

## Abstract

AI must understand human limitations to provide good service and safe interactions. Standardized data on human limits would be valuable in many domains but is not available. The data science community has to work on collecting and aggregating such data in a common and widely available format, so that any AI researcher can easily look up the applicable limit measurements for their latest project.

## Teaser

AI must understand human limitations to provide good service and safe interactions. Standardized data on human limits would be valuable in many domains but is not available. Data science community has to work on collecting and aggregating such data in a common and widely available format, so any AI researcher can easily look up applicable limit measurements for their latest project.

## Main Text

### Introduction to Artificial Stupidity

In “Computing Machinery and Intelligence,”^1^ Turing exposes common fallacies when arguing that a machine cannot pass the Turing Test. In particular, he explains why the belief that “the interrogator could distinguish the machine from the man simply by setting them a number of problems in arithmetic” because “the machine would be unmasked because of its deadly accuracy” is false. Indeed, the machine “would not attempt to give the right answers to the arithmetic problems. It would deliberately introduce mistakes in a manner calculated to confuse the interrogator.” Thus, the machine would hide its super-human abilities by giving a wrong answer, or simply saying that it could not compute the answer.

Artificial Intelligence has achieved super-human performance in some tasks, such as arithmetic or games; in this article we argue that sometimes AI’s ability might need to be artificially constrained. Such deliberate limiting is called *Artificial Stupidity*. By limiting an AI’s ability to achieve a task, to better match humans’ ability, an AI can be made safer, in the sense that its capabilities will not exceed humans’ capabilities by several orders of magnitude.. The general trend here is that AI tends to quickly achieve super-human level of performance after having achieved human-level performance. For instance, for the game of Go, in a few months, the state-of-the-art went from strong amateur, to weak professional player, to super-human performance. From that point onward, to make the AI pass a Turing Test, or make it behave human-like, AI designers must deliberately limit its capabilities.

### The Cognitive Limits of the Human Brain

Although the precise limits of human cognition are not fully known, specific recommendations on minima or maxima for different capabilities can be given.

#### Long-Term Memory

The storage capacity of the brain is generally considered to be within the bounds given by Turing^1^ (resp. 10^10^ and 10^15^ bits). Although the encoding of information in our brains is different from the encoding in a computer, we observe many similarities. To estimate the storage capacity of the human brain, we first evaluate the number of synapses available in the brain. The number of synapses in the brain has been estimated^2^ to be around 10^14^. Assuming one synapse is equivalent to one bit of information, this would give us a storage capacity of 10^14^ bits. However, such estimates are still approximate because neuroscientists do not know precisely how synapses actually encode information: some of them can encode multiple bits by transmitting different strengths, and individual synapses are not completely independent.

#### Processing

Even though the brain can encode terabits of information, humans are in practice very limited in the amount of information we can process. In his classic article,^3^ Miller showed how our minds could only hold about 7 ± 2 concepts in our working memory. More generally, three essential bottlenecks were shown to limit information processes in the brain: the Attentional Blink (AB) limits our ability to consciously perceive, the Visual Short-Term Memory (VSTM) our capacity to hold in mind, and the Psychological Refractory Period (PRP) our ability to act upon the visual world. In particular, the brain takes up to 100 ms to process complex images.^4^ Moreover, the processing time seems to take longer when the choice to make takes complex information as input. This is known as *Hick’s Law*:^5^ the time it takes to make a choice is linearly related to the entropy of the possible alternatives.

#### Computing

One approach to evaluate the complexity of the processes happening in the brain is to estimate the maximum number of operations per second. Some estimates suggest that to replicate all of a human’s function as a whole one would need about 100 million MIPS (Millions of Instructions per Second) by comparing it to the computational needs for edge extraction in robotics. Using the same estimation for the number of synapses in the brain (estimated by Turing^1^), Bostrom^2^ concludes that the brain uses at most about 10^17^ operations per second.

#### Clock Speed

The brain does not operate with a central clock. That’s why the term “clock speed” does not accurately describe processes happening in the brain. However, it is possible to compare the transmission of information in the brain to that inside a computer. Processes emerge and dissolve in parallel in different parts of the brain at different frequency bands: theta (5–8 Hz), alpha (9–12 Hz), beta (14–28 Hz) and gamma (40–80 Hz). Comparing computer and brain frequencies, Bostrom notes that “biological neurons operate at a peak speed of about 200 Hz, a full seven orders of magnitude slower than a modern microprocessor (∼2 GHz).”^6^ It is important to note that clock speed, alone, does not fully characterize the performance of a processor. Furthermore, the processes happening in the brain use several orders of magnitude more parallelization than modern processors.

### Recommendations to Build a Safer AI

Humans have clear computational constraints (memory, processing, computing, and clock speed). An Artificial General Intelligence (AGI) is not *a priori* constrained by such computational and cognitive limits. Hence, if humans do not deliberately limit an AGI in its hardware and software, it could become a *superintelligence*, i.e., an "intellect that greatly exceeds the cognitive performance of humans in virtually all domains of interest,”^6^ and humans could lose control over the AI. In this section, we discuss how to constrain an AGI to be less capable than an average person, or equally capable, while still exhibiting general intelligence. In order to achieve this, resources such as memory, clock speed, or electricity might be restricted. However, intelligence is not just about computing. Bostrom distinguishes three forms of superintelligence: speed superintelligence (“can do all that a human intellect can do, but much faster”), collective superintelligence (“A system composed of a large number of smaller intellects such that the system’s overall performance across many very general domains vastly outstrips that of any current cognitive system”), and quality superintelligence (“A system that is at least as fast as a human mind and vastly qualitatively smarter”).^6^ A hardware-limited AI could be human-level intelligent in speed, but remain qualitatively superintelligent.

#### Hardware

To begin with, we focus on how to avoid speed superintelligence by limiting the AI’s hardware. For instance, its maximum number of operations per second can be bounded by the maximum number of operations a human does. Similarly, by limiting its RAM (or anything that can be used as a working memory), we limit its processing power to process information at a rate similar to humans. Focusing only on limiting the hardware is nonetheless insufficient. We assume that, in parallel, there exist other limitations (in software) that prevent the AI from becoming qualitatively superintelligent, upgrading its hardware by changing its own physical structure, or just buying computing power online.

#### Storage Capacity

We estimated the storage capacity of the human brain to be at most 10^15^ bits, using one bit per synapse. To have a safe AGI, one should rather use much less storage capacity. For instance, Turing^1^ estimated 10^7^ bits, or 10Mb, to be a practical storage capacity to pass the Turing Test (and therefore attain AGI). Even if this seems very low, consider that an AGI could have a very elegant data structure and semantics that could allow it to store information much more concisely than our brains. In comparison, English Wikipedia in compressed text is about 12 Gb and is growing at a steady rate of 1 Gb/year. For this reason, allowing more than 10 Gb of storage capacity is unsafe. With 10 Gb of storage an AGI could have permanent access to an offline version of Wikipedia and be qualitatively superintelligent in the sense that it would have direct access to the world's most complete encyclopedia of human knowledge.

#### Memory Access

In Blum’s Human-Model,^7^ memory can be modeled as a two-tape Turing machine: one for long-term memory, one for short-term memory. Blum considers potentially infinite tapes, but for our purpose, we can consider the tapes to be at most the size discussed previously for memory (e.g., 10 Mb). According to Miller’s magical number 7 ± 2,^3^ human working-memory works with a limited amount of chunks. So, our two-tape Turing Machine should have a very short “short-term memory” tape, containing at most two or three 64-bit pointers pointing to chunks in the long-term memory (the other tape). More specifically, storing information in the long-term memory is slow, but reading from long-term memory (given the correct pointer) is fast. In modern computers, RAM’s bandwidth is about 10 GB/s, hard disk storage bandwidth is 100 MB/s, and with high clock rate a CPU can process about 25 GB/s. In order to build a safer AGI, the memory access for the two mentioned tapes must be restricted, so that we are sure that the data is being retrieved slower than by humans.

#### Processing

We previously stated how the human brain can only process a limited amount of information per second. In addition to a limited number of chunks in working memory, other features must also be implemented to slow down an AGI and make it human-level intelligent. For instance, one could introduce some artificial delay period in processing information. The length of this delay should depend on the content type. We already commented on the necessary duration of 100 ms to process complex images.^4^ Similarly, the amount of time to process a certain image might depend on the complexity and size of the image.

#### Clock Speed

As we mentioned, the brain parallelizes much more, using a totally different computing paradigm than the von Neumann architecture. Therefore, using a clock rate close to the frequency of the brain (∼10 Hz) is not relevant to our purpose, and it might prove difficult to build an AGI that exhibits human-level intelligence in real time using such a low clock rate. To solve this, one possibility is to first measure better the trajectory of thoughts occurring in the brain and then give a precise estimate of how frequently the processes in the brain are refreshed (i.e., evaluating some kind of clock rate). Another solution is to abandon the von Neumann architecture and build the AGI with a computer architecture more similar to that of the human brain.

#### Computing

In the section The Cognitive Limits of the Human Brain, we mentioned Bostrom’s estimate^2^ of at most 10^17^ operations per second for the brain. This is a very large number and could only happen if the AGI’s hardware allowed that much computing power. This will not be the case, according to what we said previously in Storage Capacity and Memory Access. More importantly, even if we could measure a number of operations per second, that would actually be lower than any number of operations per second a human brain does for any given task, it might not be a correct bound. Why? The brain has evolved to achieve some very specific tasks, useful for evolution, but nothing guarantees that the complexity or the processes happening in the brain are algorithmically optimal. Thus, the AGI could possess a structure that would be far more optimized for computing than the human brain. Therefore, restricting the number of operations alone is insufficient: the algorithmic processes and the structure of the AGI must be precisely defined so it is clear that the resulting processes happening are performing tasks at a lower rate than humans.

### Conclusions

In order to implement Artificial Stupidity limitations on AI it is first necessary to understand what the limits of human cognition are.^8^ An AI must formally understand human limitations to provide good service and safe and secure interactions. It is impossible for AI to align with human values without complete understanding of the human cognitive model. Standardized data on human limits would be extremely valuable in many domains but is not currently available for many tasks, and what is known is not conveniently available in a single repository. It is our hope that this article inspires the data science community to work on collecting and contributing such data and aggregate it in a common and widely available format.
